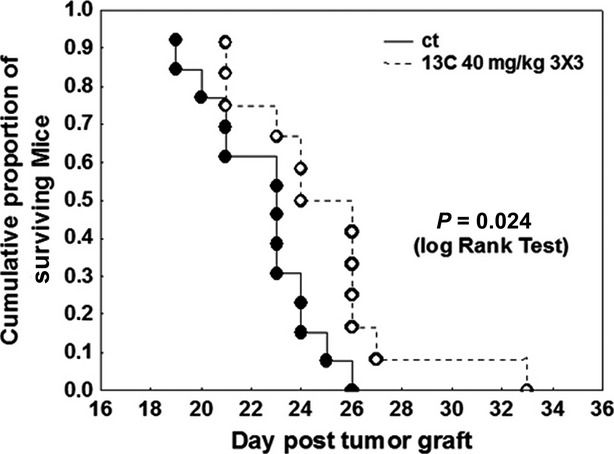# Erratum

**DOI:** 10.1111/jcmm.12640

**Published:** 2015-07-14

In 1, the authors noticed an error in Figure 8F. While the Materials and Methods and the legend to Figure 8F of the original manuscript indicated 11 mice per experimental group, Figure 8F indicated 10 mice per experimental group. In fact there were 15 mice in the control (ct) group and 16 mice in the 13C-treated one. However, the corrected Figure 8F (shown below) only includes 13 mice in the control (ct) group and 12 in the 13C-treated one. Thus, 2 and 4 mice are missing in the corrected Figure 8F with respect to the control and 13C-treated groups, respectively. The reasons why the authors did not include the 2 and the 4 missing mice in the control and the 13C-treated groups of the corrected Figure 8F are as follows. With respect to the control group, the 2 missing mice from the corrected Figure 8F include 2 mice that were euthanized 35 days after the tumor grafting procedure (while the 13th mouse of the control group in Figure 8F died on day 26 post-tumor grafting). The macroscopic analysis of these two B16F10 melanoma-grafted long-surviving mice revealed the absence of any tumor in their lungs, while the presence of this type of tumor is easily observable at the macroscopic level (Fig. 8E). This means that these 2 mice did not develop B16F10 lung pseudo-metastases, this is the reason why the authors estimated that they were not eligible for the survival analysis. The inhalation-related lung delivery of 13C necessitates about half an hour of anesthesia for the mice and 4 13C-treated mice died from anesthesia at days 9 (2 mice), 14 (1 mouse) and 16 (1 mouse). This means that these 4 mice did not wake up from anesthesia and the authors therefore estimated that they were not eligible for the survival analysis. There is no statistically significant difference between the data presented in the original Figure 8F and those in the corrected Figure 8F, meaning that the conclusions drawn from the original data in Figure 8F remain valid for the data from the corrected Figure 8F.

The authors wish to apologise for any misunderstanding or inconvenience caused.